# Idiopathic juvenile osteoporosis: a cross-sectional single-centre experience with bone histomorphometry and quantitative computed tomography

**DOI:** 10.1186/1546-0096-11-6

**Published:** 2013-02-19

**Authors:** Justine Bacchetta, Katherine Wesseling-Perry, Vicente Gilsanz, Barbara Gales, Renata C Pereira, Isidro B Salusky

**Affiliations:** 1 David Geffen School of Medicine, Division of Pediatric Nephrology, University of California, 10833 Le Conte Boulevard, Los Angeles, CA, USA; 2 Department of Radiology, Hospital Los Angeles, Los Angeles, CA, USA

**Keywords:** Idiopathic juvenile osteoporosis, Bone biopsy, Histomorphometry, QCT

## Abstract

**Background:**

Idiopathic juvenile osteoporosis (IJO) is a rare condition of poorly understood etiology and pathophysiology that affects otherwise healthy children. This condition is characterized clinically by bone pain and vertebral fractures; spontaneous recovery is observed after puberty in the majority of cases. Although decreased trabecular bone turnover has been noted previously, cortical and trabecular bone characteristics as determined by quantitative computed tomography (QCT) and their relationship to bone histomorphometry are unknown.

**Methods:**

All children with a clinical diagnosis of IJO who were followed in our center since 1995 and who had undergone at least one diagnostic bone biopsy were included in this cross-sectional analysis.

**Results:**

Fifteen patients (11 males/4 females) with median ages of 5.8 and 10.2 years at first symptoms and at referral, respectively, were included in the analysis. Histomorphometric analysis demonstrated decreased trabecular bone turnover (BFR/BS) in the majority of patients with heterogeneous parameters of trabecular mineralization and volume. QCTresults demonstrated that bone mineral density (BMD) was reduced in both trabecular/lumbar and cortical/femoral bone: Z score: -2.1 (−3.6;–1.0) and −0.9 (−8.2;1.4)in the two compartments, respectively. In the eight patients who underwent both bone biopsy and QCT, cortical BMD was associated with trabecular separation and with trabecular bone formation rate (r = 0.898 and −0.881, respectively, both p < 0.05).

**Conclusions:**

This series confirms that IJO is characterized by impaired trabecular architecture that can be detected by both bone biopsy and QCT. The association between bone biopsy and QCT results may have implications for diagnosis, treatment, and follow-up of these children.

## Background

Osteoporosis, a well-known and frequent condition in postmenopausal women, rarely affects children but may be seen in primary genetic conditions involving the skeleton or the connective tissue, such as for example OsteogenesisImperfecta, Bruck syndrome, Osteoporosis Pseudoglioma Syndrome, Ehlers-Danlos syndrome, Marfan syndrome and Homocystinuria, or may be secondary to chronic inflammation, glucocorticoid therapy, and prolonged immobilization [1]. In contrast to these relatively well defined pathologies, the pathophysiology of idiopathic juvenile osteoporosis (IJO), a rare condition characterized clinically by the insidious onset of bone pain followed by vertebral compression and long bone fractures, remains unknown.

The diagnosis of IJO is made clinically. Children of both genders present two to three years before the onset of puberty with an insidious onset of pain in the back and lower limbs in association with vertebral compression fractures, trouble ambulating and long bone fractures, especially around the weight-bearing joints [1], in the absence of family history of childhood bone disease, extra-skeletal manifestations, or growth impairment [2]. Although bone histomorphometry is the gold standard for the diagnosis of skeletal pathology, bone histomorphometric features of IJO have been characterized in only one series of 9 patients with IJO; this series reported a decrease intrabecular bone volume, thickness and number with impaired trabecular bone turnover but without any alterations in cortical bone [3,4].

Osteopenia is evident on radiographs; however, the true extent of osteoporosis and osteopenia in patients with IJO is unknown. Indeed, previous studies have evaluated bone density using dual energy X-ray absorptiometry (DXA), a technique that does not discriminate between cortical and trabecular bone density and whose assessment of bone mineral density as an area BMD (g/cm^2^) are affected by bone size, body size and composition [5]. By contrast, Quantitative Computed Tomography (QCT), a three dimensional technique based on standard body CT scanners, separately measures trabecular and cortical volumetric bone mineral densities (BMD) at central sites (lumbar spine and hip), thus permitting the accurate evaluation of bone size, geometry, and density in both trabecular and cortical bone compartments [6]. Although this technique has the potential to provide accurate assessment of cortical and trabecular bone density in the IJO population, QCT has never been evaluated in IJO patients, and never compared to the reference standard, bone biopsy. The aim of this study was therefore to report a single-centre experience of IJO in a cohort of 15 patients with at least one bone biopsy, using both histomorphometry and a non-invasive three-dimensional bone imaging technique, i.e., QCT.

## Methods

All patients with a diagnosis of IJO who were followed in our center from 1995 through 2010 and who had undergone at least one bone biopsy for clinical diagnosis were included in this retrospective descriptive analysis. This study was approved by the UCLA Human Subject Protection Committee.

The diagnosis of IJO was based on the following criteria: clinical symptoms of bone disease in a previously healthy child (e.g., past history of fractures, osteopenia, bone pain), evidence of low bone mass on BMD assessed by QCT and/or of osteoporosis on bone histomorphometry, without any evidence for a secondary osteoporosis or other primary bone disease. A clinical score was developed which accounted for the subjective severity of disability (moderate or severe restriction of daily activities) and the objective number of fractures. Children were divided into two groups based on number of fractures: moderate disability was defined as fewer than five fractures (clinical score 1) while severe disability was defined as 5 or more fractures (clinical score 2).

QCT examination was performed at three sites (both femora and the spine) using the same scanner (General Electric Hilite Advantage, Milwaukee, WI) in all patients. Measurements of the size (i.e., cross-sectional area, CSA) and the density of cortical bone were obtained from a 1.5 mm-thick sections at the midshaft of the femora at 120 kVp and 70 mA; the final results consisted in the mean of both femora. Measurements of the size (i.e., cross-sectional area CSA) and the density of cancellous bone were obtained from 10 mm-thick imaging sections at the mid-portions of the first four lumbar vertebrae at 80 kVp and 70 mA; the final results consisted in the mean of L1, L2, L3 and L4 values. Reference values assessed with QCT are 2000 ± 60 mg/cm^3^for cortical BMD in all children, and 250 ± 45 and 285 ± 45 mg/cm^3^ for cancellous BMD in pre-pubertal and pubertal children, respectively. These local reference values are routinely used by Dr. Gilsanz, and trabecular BMD details have been described from a cohort of 1222 White healthy subjects aged 5–21 years [7]. Z-scores were calculated with the following formula: Z-score = (Result of the patient – Mean of the control population)/SD of the control population. The radiation exposure per measurement was 1 mSv at the spine and 1.5 mSv at the femur. The variation in this technique is less than 1.5% in children [8].

The full thickness bone biopsy, performed at the iliac crest 2 to 5 days after the end of a double tetracycline labeling (10–15 mg/kg per day, taken orally three times a day during two 2-day periods separated by a 10 to 12-day free interval), was performed as previously reported [9]. Biopsy specimens 0.5 cm in diameter by 1 to 2 cm in length were fixed in 10% phosphate-buffered formalin for 48 hours, then dehydrated in alcohol, cleared with xylene, and embedded in methylmethacrylate. Static histomorphometric parameters were evaluated in lamellar portions of undecalcified 5 μm sections treated with Goldner stain and tetracycline labeling was assessed in unstained 10 μm sections of lamellar bone, by the same observer (RP). Bone histology was reported using the terminology established by the Nomenclature Committee of the American Society of Bone and Mineral Research [10]. Derived indices were calculated by additional formulas, as detailed previously [11]. Normal pediatric values for all histomorphometric parameters were previously obtained from double-tetracycline-labeled iliac crest bone biopsies from 31 pediatric controls (mean age 12.4 ± 8.9 years, 71% males, 48% Caucasian, 26% Hispanic) who were undergoing elective orthopedic surgery [12,13].

Statistical analysis was performed using the SPSS software® 18.0 for Windows. Comparisons between groups were performed using the nonparametric Wilcoxon signed rank test. Bivariate correlations were computed with the Spearman test. Comparisons of percentages were made with a chi-square test. All statistical tests were performed at the two-sided 0.05 level of significance. Results are presented as median (range).

## Results

Fifteen patients (11 males/4 females) with at least one bone biopsy were included in the analysis; only 12 initial bone biopsies were interpretable as one biopsy contained growth plate and two were technically unreadable. Eleven patients had an initial QCT. Eight patients underwent both baseline QCT and bone biopsy.

### Patients

The median (range) for age at first symptoms and for age at referral were 5.8 (1.5–14) and 10.2 (5.5-15.4) years, respectively. The median time between first symptoms and the diagnosis of IJO was 3.5 (0.2-9.4) years. The median (range) percentile of height at referral was 88 (15–98); 11 patients were at or above the 50^th^ percentile for height at diagnosis. Twelve patients were White, two were Hispanic and one was White by her father and Hispanic by her mother.

Most of the patients had a family history of bone disease or disorder of calcium/phosphate metabolism, including post-menopausal osteoporosis in four, scoliosis in one, and parathyroid adenoma in one. Two patients were siblings and two others were homozygous twins. Ten patients underwent fibroblast analysis or direct gene analysis to rule out Osteogenesis Imperfecta. All but one patient had a history of fractures occurring with minimal trauma; five complained of back pain and four of leg pain. Two patients had kyphosis or scoliosis, two had vertebral compression fractures and one developed avascular necrosis and osteomyelitis. Of note, one patient received calcitonin prior to referral; even though calcitonin is known to have inhibitory effects on osteoclast activity [14,15], it is difficult to evaluate in which extend this treatment could have attenuated the severity of the bone impairment at baseline. Table 1 summarizes the main clinical characteristics of the patients.

**Table 1:** Main clinical characteristics of the 15 patients with IJO

	**Sex**	**Familial bone history**	**Genetic or skin analysis**	**Clinical score**	**Age first referral (years)**	**Age first symptom (years)**	**Time between first symptom and diagnosis (years)**	**Centile-size at diagnosis**	**Main initial symptom**	**Back pains**	**Legs pains**	**Chest**	**Vertebral compression fracture**	**Other symptoms**
**1**	M	3 fractures mother, brother of pt2	Negative	2	15.4	6.0	9.4	90	fracture	No	No	No	No	Increased urinary NTX
**2**	F	Osteoporosis mother, sister of pt1	NP	1	13.5	5.0	8.5	45	back pain	Yes	No	No	No	Transient hyperPTH
**3**	M	No	Negative	2	8.2	2.5	5.7	95	fracture	No	No	No	No	No
**4**	M	Osteoporosis grand-mother	Negative	2	14.3	14.0	0.3	85	fracture	Yes	Yes	No	No	Increased urinary NTX
**5**	F	No	Negative	1	5.9	5.5	0.4	50	fracture	No	No	No	No	No
**6**	M	Osteoporosis grand-mother	NP	1	13.3	10.5	2.8	75	fracture	No	No	No	No	No
**7**	F	No	Negative	1	7.8	7.6	0.2	90	osteopenia	No	No	No	No	No
**8**	M	Rheumatoidarthritis mother	Negative	2	12.9	8.0	4.9	80	fracture	Yes	Yes	No	No	Skin hyperpigmentation, Low 25-vitD
**9**	M	Parathyroid adenoma father	Negative	2	10.2	3.5	6.7	90	fracture	No	No	No	No	No
**10**	M	Twin brother (pt 11) with fractures	Negative	2	13.9	5.0	8.9	98	fracture	No	No	No	No	No
**11**	M	Twin brother (pt 10) with fractures	Negative	1	13.9	13.0	0.9	98	fracture	No	No	No	No	No
**12**	M	Osteoporosis grand-mother	Negative	2	5.5	1.5	4.0	15	fracture	No	Yes	No	No	No
**13**	M	Scoliosis father	NP	2	8.1	5.0	3.1	95	fracture	Yes	No	Yes	Yes	No
**14**	M	No	NP	2	9.5	9.0	0.5	15	fracture	Yes	Yes	Yes	Yes	Osteomyelitis radius and avascular necrosis, Transient hypercalciuria
**15**	F	NA	NA	2	10.0	NA	NA	NA	fracture	NA	NA	NA	NA	NA

NA: non available data.

NP: not performed.

M/F: male/female.

PTH: parathyroid hormone.

### Bone histomorphometry

**Figure 1:**
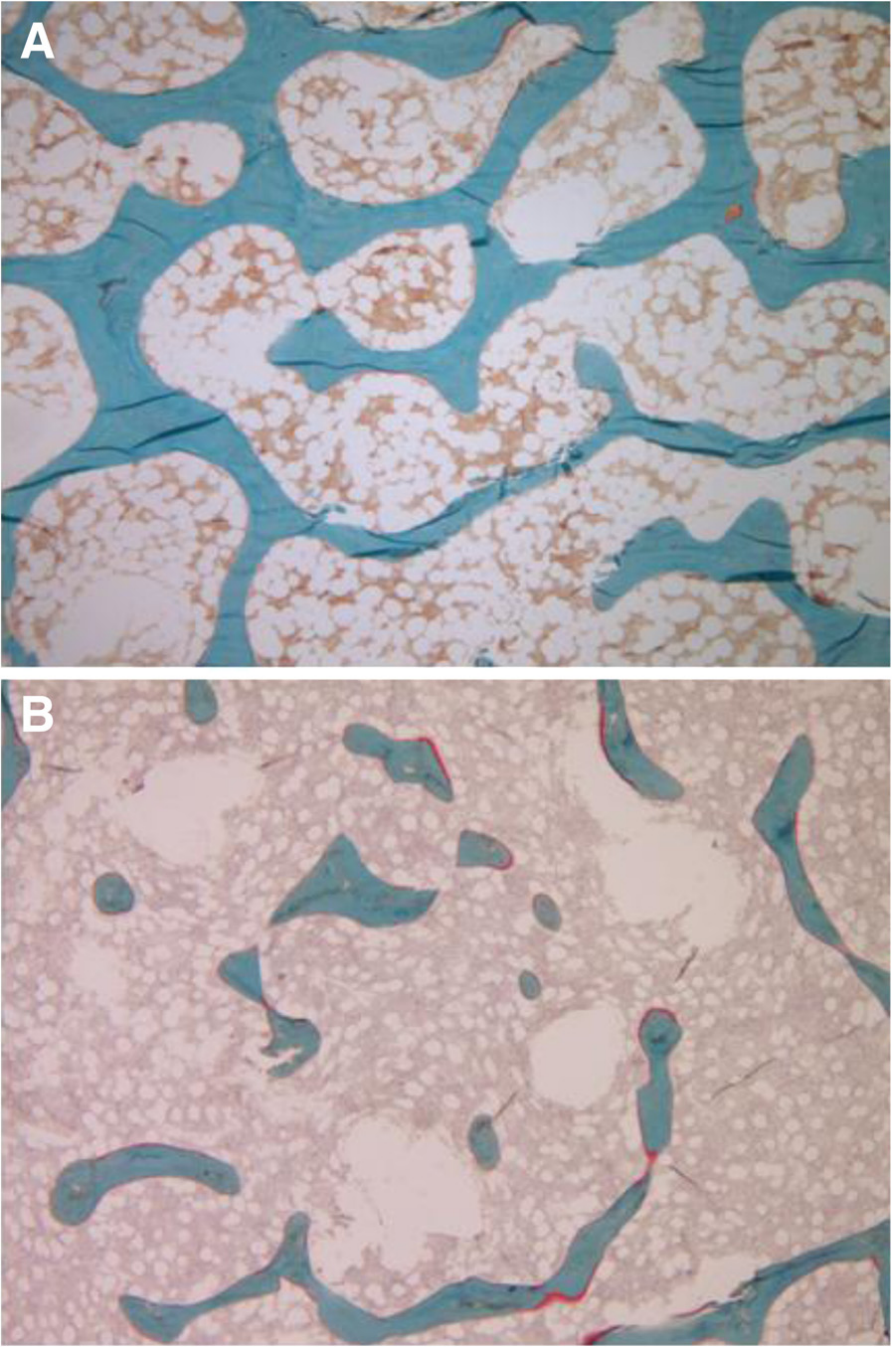
**Bone biopsy (Goldner'strichrome, magnification 40x) showing in red, the unmineralized matrix and in green the mineralized bone matrix. A**- in a healthy control: the trabecular micro-architecture and connectivity are normal. **B**- in a patient with IJO: in addition to a global thinning of the trabeculae, there are disconnections within the trabecular network.

Trabecular bone histomorphometric parameters of turnover, mineralization and volume parameters are summarized in Tables 2 and 3. Bone turnover (BFR/BS) was decreased in 5 patients while parameters of trabecular mineralization and volume were heterogeneous. Figure 1 presents morphological findings of IJO in comparison to normal age-matched bone.

**Table 2:** Baseline bone biopsies findings in 12 patients with IJO

	**Parameter**	**Median**	**Minimum**	**Maximum**	**Normal range (10–11)**
**Volume**	Trabecular separation (Tb.Sp) (μm)	432	367	610	299–587
	Trabecular number (Tb.N) (#/mm)	1.83	1.42	2.14	1.3–2.7
	Trabecular thickness (Tb.Th) (μm)	107	79	145	72–177
	Bone volume (BV/TV) (%)	19.71	13.61	26.8	9.3–22.7
**Mineralization**	Mineral apposition rate (MAR) (μm/day)	0.8	0.5	0.99	0.9–2.4
	Mineralization lag time (MLT) (days)	18.13	4.59	105.12	4.1–71.7
	Osteoid maturation time (OMT) (days)	8.68	4.11	15.57	1.9–10.4
	Osteoid thickness (O.Th) (μm)	6.92	2.73	9.61	3–13.9
	Osteoid volume (OV/BV) (%)	1.21	0.26	4.65	0.2–5.86
	Osteoid surface (OS/BS) (%)	8.69	3.15	30.39	1.4–23.96
**Turnover**	Bone formation rate (BFR/BS) (μm^2^/mm^3^/year)	12.81	1.95	78.91	10–73.4
	Eroded surface (ES/BS) (%)	4.28	0	13.27	1.6–6.84

**Table 3:** Qualitative abnormalities of bone mineralization, turnover and volume in 12 IJO patients with bone biopsies

		**High**	**Low**
Mineralization	Mineralization lag time (MLT)	1	1
	Osteoid maturation time (OMT)	4	0
	Osteoid thickness (O.Th)	0	1
	Osteoid surface (OS/BS)	3	0
	Osteoid volume (OV/BV)	0	0
Volume	Bone volume (BV/TV)	5	0
	Trabecular thickness (Tb.Th)	0	0
	Trabecular separation (Tb.Sp)	1	1
	Trabecular number (Tb.N)	0	0
Turnover	Mineral apposition rate (MAR)	0	7
	Eroded surface (ES/BS)	5	3
	Bone formation rate (BFR/BS)	2	5

### Biochemical parameters

Biochemical abnormalities were found in five patients: one displayed transient hypercalciuria, two had increased levels of urinary N-telopeptide, one was 25 (OH) vitamin D deficient (defined by a 25 (OH) vitamin D level of 22 ng/mL) and one had a moderate but transient increase in PTH (120 pg/mL). The median (range) for PTH and 25 (OH) vitamin D at the time of the bone biopsy were 26 (8–120) pg/ml and 44 (22–93) ng/ml, respectively. Serum PTH levels were directly related to trabecular bone volume (BV/TV, r = 0.670, p = 0.024), and 25 (OH) vitamin D levels were associated with the rate of bone mineralization (osteoid maturation time (OMT), r = −0.786, p = 0.036 with a similar trend for mineralization lag time (MLT), r = −0.714, p = 0.07), although not with osteoid accumulation (as measured by osteoid volume (OV/BV), osteoid thickness (O.Th), or osteoid surface (OS/BS)).

### Quantitative computed tomography (QCT)

Trabecular (lumbar) and cortical (femoral) bone mineral density were both low at 187 (124–224) mg/cm^3^ and 1946 (1506–2081) mg/cm^3^, respectively, with a Z-score of −2.1 (−3.6;–1.0) and −0.9 (−8.2;1.4), respectively. Trabecular (lumbar) and cortical (femoral) cross sectional area were 11.35 (4.72–12.69) cm^2^ and 4.81 (1.86–6.61) cm^2^, respectively; Z-score according to age and body weight for these two parameters were 2.30 (range −0.8, 4.4) and −0.7 (−3.0, 0.9), respectively. There was no significant association between cortical and trabecular bone mineral densities (r = 0.395, NS).

### QCT and bone histomorphometry

**Figure 2:**
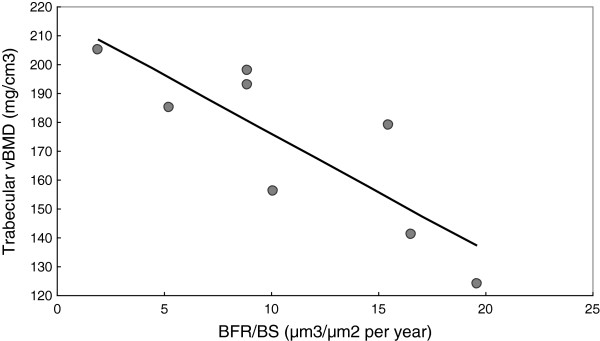
**Relationship between trabecular BMD at the spine (QCT) and BFR/BS on bone biopsy (r = −0.881, p = 0.004).** BMD: bone mineral density. QCT: quantitative computed tomography. BFR/BS: bone formation rate/bone surface.

A comparison of the eight patients who had both bone biopsy and QCT within a median time frame between the two examinations of 0.4 years (0.1–2.5) revealed a relationship between cortical bone mineral density and parameters of trabecular bone volume (trabecular number (Tb.N): r = 0.898, p = 0.02; trabecular separation (Tb.Sp): r = −0.838, p = 0.009), with a trend between cortical bone mineral density and cortical bone volume (external bone volume (BV/TV) and external porosity, r = 0.754 and −0.754, respectively, both p = 0.084). Trabecular bone mineral density was associated with trabecular bone turnover (trabecular bone formation rate (BFR/BS): r = −0.881, p = 0.004). Figure 2 illustrates the relationship between trabecular bone mineral density and BFR/BS. Table 4 summarizes the QCT and bone biopsy results per patient.

**Table 4:** Main results of bone biopsy and QCT per patient

**Pt**	**Age biopsy (years)**	**BV/TV (%)**	**OS/BS (%)**	**BFR/BS (μm**^**2**^**/mm**^**3**^**/year)**	**Mlt (days)**	**Age QCT (years)**	**Trabecular BMD (mg/cm**^**3**^**)**	**Trabecular Z-score**	**Cortical BMD (mg/cm**^**3**^**)**	**Cortical Z-score-**
**1**	15.5	21.24	3.14	5.72	9.52					
**2**	14.6	20.27	9.00	5.23	36.34	14.5	185	−2.22	1946	−0.90
**3**	8.2	18.12	7.14	15.51	4.59	12.8	193	−2.04	2081	1.35
**4**	14.6	26.15	30.39	78.91	13.52					
**5**	6.0	17.42	7.38	1.95	105.12	6.3	205	−1.00	1981	−0.32
**6**	13.8					13.6	224	−1.36	1988	−0.20
**7**	8.6	16.96	25.63	15.62	28.77					
**8**	12.9	20.52	8.38	19.60	11.27	11.8	124	−3.58	1934	−1.10
**9**	10.5	19.13	12.46	26.93	11.22	15.9	201	−1.87	1898	−1.70
**10**	13.9	18.12	7.70	8.91	22.74	14.1	198	−1.93	1916	−1.40
**11**	13.9					14.1	187	−2.18	2015	0.25
**12**	5.7	13.60	4.88	10.11	6.07	5.5	156	−2.09	1506	−8.23
**13**	8.3	23.61	13.23	16.52	23.30	7.9	141	−2.42	1916	−1.40
**14**	13.8					9.5	143	−2.38	1950	−0.83
**15**	10.0	26.79	19.34	9.41	58.33					

### Clinical severity score

The proportion of boys with clinically severe idiopathic juvenile osteoporosis was higher than the proportion of boys with clinically moderate disease. There were no imaging differences between the two groups to account for the clinical findings; however, patients with severe osteoporosis had significantly lower mineralization lag time (MLT): the median (range) for MLT was 36.3 (28.8–105.1) and 12.4 (4.6–58.3) days in moderate and severe osteoporosis, respectively (p = 0.028).

## Discussion

This study is the first to compare QCT, which allows separate measurements of cortical and trabecular bone mineral density, with bone biopsy, the reference standard for evaluating bone, in a cohort of 15 children with idiopathic juvenile osteoporosis. Major findings include the description of decreased bone turnover in many patients, a decrease in both trabecular and cortical Z-scores for bone mineral density, and a relationship between both cortical and trabecular bone density with parameters of bone histology, thus allowing a non-invasive evaluation of cortical and vertebral bone volume in these children.

Two large clinical cohorts of IJO patients have been previously reported, containing 21 and 61 patients respectively [16,17]. The median age at first symptoms in the current cohort was similar to these previous studies by Smith et al. [16], and Lorenc et al. [17]; however, a greater proportion of males (73%) was present in the current study with a significantly greater number of males presenting with clinically severe osteoporosis. Of note, the majority of patients in the current cohort demonstrated normal or high height percentile for age at referral; this clinical feature may distinguish idiopathic juvenile osteoporosis from other constitutional bone diseases at the time of the first evaluation.

Histomorphometric data from Rauch *et al.* have described the presence of decreased bone formation, in the absence of increased bone resorption or cortical damage, in trabecular bone of patients with idiopathic juvenile osteoporosis [4]. They thus hypothesized that the condition may be due to the presence of an apparently normal number of osteoblasts whose function is altered, leading to a decreased rate of matrix deposition [3]. In the current study, we have confirmed the findings by Rauch et al. demonstrating decreased bone turnover in the majority of patients; however, a greater variability in mineralization and volume parameters were noted in our cohort. In comparison with Rauch et al., histomorphometric parameters of trabecular bone turnover, mineralization, and volume were higher in the current cohort. These discrepancies may be explained in part by the differences between the reference values.

Published data on bone mineral density in children with idiopathic juvenile osteoporosis have been obtained from DXA. Using this technique, Lorenc et al. reported that total body bone mineral density was lower than the normal for age [17]. Although it has been widely used in children, the areal measurement of bone mineral density by DXA has at least two main limitations in children: its reliance on areal density rather than volumetric density, a parameter which is modified by growth, and its inability to distinguish between trabecular and cortical bone compartments which have different physiology and growth patterns [6,18]. More recently, Mayranpaa et al. performed DXA and bone biopsy in a cohort of 24 consecutive children (17 males, median age 12 years) presenting with idiopathic osteoporosis. They showed that histomorphometric findings correlated poorly with fracture history, circulating bone biomarkers and DXA findings; in contrast, vitamin D deficiency and low lumbar BMD were associated with high bone turnover on the biopsy [19]. In this series, we also found an association between vitamin D status and bone biopsy results, with a relationship between 25 (OH) vitamin D levels and bone mineralization. We also found a direct link between serum PTH levels and trabecular bone volume that were not described in the Finnish study.

In the current study, which used QCT to discriminate cortical and cancellous bone compartments, a decrease in both trabecular and cortical BMD Z-scores was observed, with greater impairments in trabecular BMD, confirming previous reports which highlighted the greater importance of impaired trabecular bone in patients with idiopathic juvenile osteoporosis. However, the low Z-score for both BMD and cross-sectional area in the cortical compartmentmay explain the important frequency of cortical fractures also observed in this cohort; indeed, even though children with IJO are usually known to present both vertebral compression fractures and long bone fractures, in our series, only two patients presented with vertebral compression fractures whereas long bone fractures and extremity fractures (e.g., toes, metatarse) were much more frequent; however, due to the retrospective design of the study, compression fractures were not systematically screened in the patients, but only in case of clinical symptoms. Furthermore, the association between histomorphometry and QCT parameters highlights the potential interest of this non-invasive three-dimensional imaging technique for the follow-up of children with IJO. In some patients the biopsy and QCT were not obtained at the same age due to the retrospective design of the study and may have lessened the association between QCT and bone biopsy; however, to the best of our knowledge, this association between QCT and bone biopsy has never been evaluated in pediatrics and further prospective studies are warranted to evaluate its potential clinical and therapeutic ramifications. Moreover, after the new definition in 2000 of osteoporosis by the National Institute of Health (NIH), adding ‘quality’ criteria in addition to the quantitative evaluation of BMD, new bone imaging techniques have been developed, including High-Resolution peripheral Quantitative Computed Tomography (HR-pQCT), and therefore leading to an improvement in bone evaluation. Indeed, HR-pQCT can assess both compartmental (i.e., total, cortical and trabecular) volumetric BMD and trabecular microarchitecture [6]. This technique, corresponding to an improvement of QCT, has already been validated in other pediatric populations [20,21]; it could theorically be of interest in patients with IJO and should be evaluated in the future.

Recent studies have reported an association between mutations in the low-density lipoprotein receptor-related protein 5 (LRP5) and low bone mass in children [22]; however, these data cannot fully explain the pathophysiology of juvenile osteoporosis, and the initial trigger of the decreased osteoblast performance remains to be determined. In the context of idiopathic juvenile osteoporosis, Laine et al. recently described a higher prevalence of LRP5 polymorphisms in a cohort of 27 children (14 males) [23]. Notably, they also found that one-third of the patients had at least one parent with a BMD below the expected range for age [23]. In our series of case, ten patients underwent fibroblast analysis or direct gene analysis to rule out Osteogenesis Imperfecta but other genes were not studied. We also found a positive familial history of bone disease in more than half of our patients.

Currently, no evidence-based guidelines exist for the management of idiopathic juvenile osteoporosis. Some case reports have described the potential benefit of bisphosphonate therapy [24-26]; however, few controlled studies exist in the use of bisphosphonates in children in general and in those with juvenile osteoporosis in particular and the use of bisphosphonates in this population remains controversial due to inadequate long-term efficacy and safety data. Dietary and life-style modifications, including adequate calcium and vitamin D intake, adequate exercise, and avoidance of obesity, may be adopted in all children, including those with IJO, with very little risk. Indeed, the prevalence of 25 (OH) vitamin D deficiency in the current cohort and the association between 25 (OH) vitamin D levels and parameters of skeletal mineralization suggest that early detection and treatment of vitamin D deficiency and insufficiency may be beneficial in this patient population.

## Conclusions

In conclusion, idiopathic juvenile osteoporosis is a rare bone disease affecting pre-pubertal children, with both cancellous and cortical impairments. Its etiology and pathophysiology are not well understood and its diagnosis remains one of exclusion. The current study brings new insights into this rare disease, confirming bone impairment as assessed by bone biopsy and QCT, and therefore allowing the description of a promising association between bone histology and QCT results that could be of relevance in daily practice.

## Abbreviations

BFR/BS: Bone formation rate/Bone surface; BMD: Bone mineral density; BV/TV: Bone volume/Tissue volume; DXA: Bone density using dual X-ray absorptiometry; ES/BS: Eroded surface/Bone surface; IJO: Idiopathic juvenile osteoporosis; LRP5: Lipoprotein receptor-related protein 5; MAR: Mineral apposition rate; MLT: Mineralization lag time; O.Th: Osteoid thickness; Ob.S/BS: Osteoblast surface/Bone surface; Oc.S/BS: Osteoclast surface/Bone surface; OMT: Osteoid maturation time; OS/BS: Osteoid surface/bone surface; OV/BV: Osteoid volume/bone volume; PTH: Parathyroid hormone; QCT: Quantitative computed tomography; Tb.N: Trabecular number; Tb.Sp: Trabecular separation; Tb.Th: Trabecular thickness.

## Competing interests

The study sponsors had no role in the study design; the collection, analysis, and interpretation of data; the writing of the report; and the decision No honorarium, grant, or other form of payment was given to anyone to produce the manuscript and to submit the paper for publication. The authors declare no conflicts of interest.

## Authors’ contributions

The first draft of the manuscript was written by JB and KWP. RP read the bone biopsies and VG performed the QCT analyses. BG was involved in the data’s collection. IS designed the study and revised the manuscript. All authors approved the last version of the manuscript.

## Sources of financial assistance

This work was supported in part by educational grants (*AcadémieFrançaise/Jean Walter Zellidja, RéunionPédiatrique de la Région Rhône Alpes, SociétéFrançaise de Pédiatrie/Evian, Fondation pour la RechercheMédicale*, Philippe Foundation, JB), by USPHS grants DK 67563, DK 35423, DK 080984-01A1 and funds from the Casey Lee Ball Foundation (IBS and KWP).
